# Initial reconstruction results from a simulated adaptive small animal C shaped PET/MR insert

**DOI:** 10.1186/2197-7364-2-S1-A56

**Published:** 2015-05-18

**Authors:** Nikos Efthimiou, Theodora Kostou, Panagiotis Papadimitroulas, Tsoumpas Charalampos, George Loudos

**Affiliations:** Technological Educational Institute of Athens, Greece; Department of Medical Physics, School of Medicine, Univ. of Patras, Greece; Division of Biomedical Imaging, University of Leeds, Leeds, UK

Traditionally, most clinical and preclinical PET scanners, rely on full cylindrical geometry for whole body as well as dedicated organ scans, which is not optimized with regards to sensitivity and resolution. Several groups proposed the construction of dedicated PET inserts for MR scanners, rather than the construction of new integrated PET/MR scanners. The space inside an MR scanner is a limiting factor which can be reduced further with the use of extra coils, and render the use of non-flexible cylindrical PET scanners difficult if not impossible. The incorporation of small SiPM arrays, can provide the means to design adaptive PET scanners to fit in tight locations, which, makes imaging possible and improve the sensitivity, due to the closer approximation to the organ of interest. In order to assess the performance of such a device we simulated the geometry of a C shaped PET, using GATE. The design of the C-PET was based on a realistic SiPM-BGO scenario. In order reconstruct the simulated data, with STIR, we had to calculate system probability matrix which corresponds to this non standard geometry. For this purpose we developed an efficient multi threaded ray tracing technique to calculate the line integral paths in voxel arrays. One of the major features is the ability to automatically adjust the size of FOV according to the geometry of the detectors. The initial results showed that the sensitivity improved as the angle between the detector arrays increases, thus better angular sampling the scanner's field of view (FOV). The more complete angular coverage helped in improving the shape of the source in the reconstructed images, as well. Furthermore, by adapting the FOV to the closer to the size of the source, the sensitivity per voxel is improved.

